# Molecular detection of tick-borne pathogens in canine population and *Rhipicephalus sanguineus* (*sensu lato*) ticks from southern Metro Manila and Laguna, Philippines

**DOI:** 10.1186/s13071-018-3192-y

**Published:** 2018-12-17

**Authors:** Remil L. Galay, Anna Angelica L. Manalo, Sidney Lyndon D. Dolores, Irene Pearl M. Aguilar, Kristina Andrea C. Sandalo, Kathlyn B. Cruz, Billy P. Divina, Masako Andoh, Tatsunori Masatani, Tetsuya Tanaka

**Affiliations:** 1 Department of Veterinary Paraclinical Sciences, College of Veterinary Medicine, University of the Philippines Los Baños, 4031 Los Baños, Laguna Philippines; 2 College of Veterinary Medicine, Cagayan State University, Carig, 3500 Tuguegarao City, Cagayan Philippines; 3 Laboratory of Public Health, Joint Faculty of Veterinary Medicine, Kagoshima University, 1-21-24 Korimoto, Kagoshima, 890-0065 Japan; 4 Transboundary Animal Diseases Research Center, Joint Faculty of Veterinary Medicine, Kagoshima University, 1-21-24 Korimoto, Kagoshima, 890-0065 Japan; 5 Laboratory of Infectious Diseases, Joint Faculty of Veterinary Medicine, Kagoshima University, 1-21-24 Korimoto, Kagoshima, 890-0065 Japan

**Keywords:** Canine tick-borne pathogens, *Rhipicephalus sanguineus* (*sensu lato*), Philippines, Southeast Asia

## Abstract

**Background:**

The tropical climate of the Philippines and the high population of dogs, particularly in cities, favors the life-cycle of the brown dog tick, *Rhipicephalus sanguineus* (*sensu lato*), a vector of several canine tick-borne pathogens (TBPs) including zoonotic *Rickettsia* spp. Suspected cases of infections are commonly encountered in veterinary clinics, but the specific TBPs are rarely identified. Furthermore, infection with *Rickettsia* is not being clinically examined in dogs. In this study, the occurrence of TBPs in blood and ticks collected from household and impounded dogs in highly populated areas of the Philippines, Metro Manila, and the nearby province of Laguna, was examined.

**Results:**

A total of 248 blood samples and 157 tick samples were subjected to PCR. First, samples were screened using primers for *Anaplasma*/*Ehrlichia* spp. and *Babesia*/*Hepatozoon* spp. Those that turned positive were further subjected to species-specific PCR. *Rickettsia* spp. were also detected through a nested PCR. Of the 248 blood samples, 56 (22.6%) were positive for *Anaplasma*/*Ehrlichia* spp., while 19 (7.6%) were positive for *Babesia*/*Hepatozoon* spp. Species-specific PCR revealed that 61 (23.4%) had a single TBP, with *Ehrlichia canis* being detected in 39 (15.7%) dogs, while 14 (5.6%) dogs were positive for different combinations of two to four TBPs. *Rickettsia* infection was detected in 6 (2.4%) dogs. In tick samples, 8 (3.2%) were positive for *Ehrlichia*/*Anaplasma* spp., while only 1 (0.63%) was positive for *Babesia*/*Hepatozoon* spp. As in the blood samples, *E. canis* was the most detected, being found in 5 (2%) samples. No tick samples tested positive for *Rickettsia* spp.

**Conclusion:**

*Ehrlichia canis* is the most common TBP affecting dogs in the Philippines. Co-infection with TBPs is quite common, hence testing for multiple TBPs is necessary. Through nested PCR, *Rickettsia* infection was detected in dogs, and to the authors’ knowledge, this study provides the first molecular evidence of *Rickettsia* infection in dogs in the Philippines.

## Background

Ticks are known to be capable of transmitting more pathogens to humans and animals than any other arthropod [1]. Tick-borne pathogens (TBPs) cause critical infections that are potentially fatal. The incidence of tick-borne diseases (TBDs) has been reported to have increased worldwide in recent years, seriously threatening human and animal health. As with other vector-borne diseases, the complex epidemiology of TBDs makes control difficult [2]. Knowledge of the occurrence and distribution of TBPs in human and animal populations, as well as in the tick vector in various geographical areas, is critical for their control.

The brown dog tick *Rhipicephalus sanguineus* is the most widely distributed tick, prevalent throughout the year in tropical and subtropical areas [3]. It is known to be a vector of several canine pathogens including rickettsiae *Ehrlichia canis* and *Anaplasma platys* and protozoans *Babesia vogeli* and *Hepatozoon canis*, as well as zoonotic *Rickettsia* species [4]. *Rhipicephalus sanguineus* is a three-host tick, dropping from its host after each blood meal and molting in the environment to the next stage. Thus, this tick can utilize a different host for every blood meal, and therefore has a higher chance of spreading pathogens it might carry to other hosts. A single tick can be a vector for several pathogens and can transmit those pathogens simultaneously in a single blood meal [5]. Thus, concurrent infection with different TBPs can occur in dogs in endemic areas, especially in dogs heavily infested with ticks.

Among the common tick-borne bacterial diseases of dogs in Southeast Asia are canine monocytic ehrlichiosis (CME) caused by *E. canis* and canine infectious cyclic thrombocytopenia caused by *A. platys.* CME causes more severe clinical signs, but the common feature of these two diseases is thrombocytopenia [6]. Meanwhile, the tick-borne protozoan parasites of dogs are *Babesia* and *Hepatozoon* species, the latter being transmitted when the tick vector is ingested by the dog rather than through the tick’s blood meal as all the other TBPs. Anemia is a common finding in uncomplicated babesiosis and *H. canis* infection [7, 8]. Additionally, several *Rickettsia* species belonging to the spotted fever group can infect dogs. These include *R. rickettsii* (the cause of Rocky Mountain spotted fever), *R. conorii* (the cause of Mediterranean spotted fever), *R. parkeri*, and *R. massiliae*; all of these are known to be zoonotic [9]*. Rickettsia rickettsii* causes a potentially fatal disease in dogs, while the disease due to other species are either rarely reported or unknown [9].

The tropical climate of Southeast Asian countries, which include the Philippines, the presence of stray or neglected companion animals, and the high popularity of dog ownership all contribute to favorable conditions for tick survival and reproduction, leading to enhanced transmission of TBPs [10]. The growing popularity of dog ownership in the Philippines resulted in an increased dog population particularly in big cities, including the country’s capital region, Metro Manila, and the nearby province of Laguna. In many communities, dogs are allowed to roam freely outdoors. Throughout the year, veterinarians in these areas encounter dogs showing clinical manifestations suggestive of TBDs, which include bleeding tendencies, anemia, and thrombocytopenia. CME or babesiosis is usually suspected in those cases; however, the specific etiologic agent is rarely identified due to limitations of the diagnostic tests performed. Moreover, the occurrence of *Rickettsia* infection in dogs and dog ticks is not examined. Thus, the Philippines still lacks a moderate amount of epidemiological data regarding the geographical occurrence of TBPs. There are several reports on detection of TBPs such as *A. platys*, *E. canis*, *B. vogeli* and *H. canis* using PCR in some parts of the country, such as Cebu [11–13], Nueva Ecija [14], and some parts of Metro Manila [15, 16], but these studies examined only a small number of pet dogs within small geographical locations. Here we investigated the occurrence of the commonly reported canine TBPs and *Rickettsia* spp. in household dogs presented to veterinary clinics and hospitals and in impounded dogs, as well as in *R. sanguineus* (*sensu lato*) ticks collected from those dogs, from 12 areas in Metro Manila and Laguna, Philippines.

## Methods

### Geographical area, study population and sample collection

**Fig. 1:**
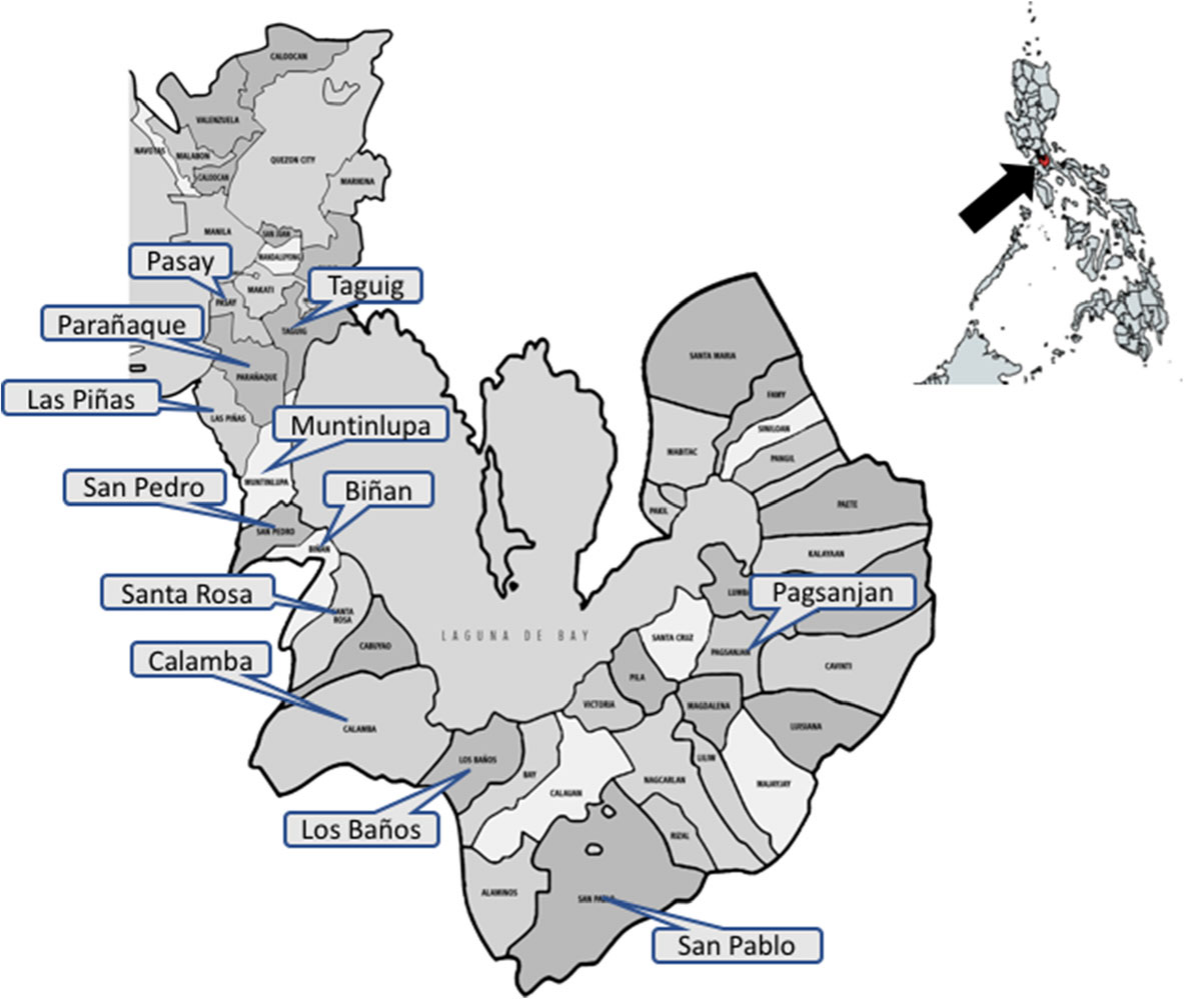
Map of the area of study in the Philippines. The arrow in the smaller map of the Philippines indicates the location of Metro Manila and Laguna. The enlarged map of Metro Manila and Laguna shows the 12 cities and municipalities (labelled) from which the samples were obtained

This study included cities in the southern part of Metro Manila (Pasay, Taguig, Parañaque, Las Piñas and Muntinlupa) and cities or large municipalities in Laguna (San Pedro, Biñan, Santa Rosa, Calamba, Los Baños, San Pablo and Pagsanjan) located between 14°3'N and 14°32'N, and 121°0'E and 121°27'E (Fig. 1). Samples of blood and, if present, ticks were collected from pet dogs and impounded stray or abandoned dogs regardless of breed, age and sex. Samples from household dogs were obtained in cooperation with veterinary clinics and hospitals located in the selected cities and municipalities, while a samples from stray or abandoned dogs were obtained from a dog pound in Laguna. The selection criteria for the dogs were: (i) a recent history of or existing tick infestation; (ii) the presence of clinical signs suggestive of tick-borne infection; (iii) those previously suspected to have any tick-borne disease and currently undergoing treatment; and (iv) those that have apparently recovered from any tick-borne disease. A questionnaire was used to obtain pertinent information on the dogs, including the breed, sex, age, chief complaint upon clinical presentation, medical history, vitals and presenting clinical signs if any. The nature of confinement (i.e. indoor or outdoor), presence of other dogs in the household, and medications given prior to clinical presentation were also noted.

The veterinarians in participating veterinary clinics and hospitals agreed to collect the blood and tick samples, while veterinary students collected samples from dogs in the dog pound. At least 0.5 ml of blood was collected from each dog using a 3 ml syringe with a 23G needle and then placed in a 1.5 ml sterile EDTA coated tube. Each dog was also inspected for ticks and if present, 2–5 partially fed to fully engorged ticks were manually detached and placed in 1.5 ml tubes. All samples were kept in a freezer (-20 °C) in the clinics or hospitals until retrieval for processing in the laboratory.

### Tick identification and pooling

Ticks were examined under a stereomicroscope and identified based on morphology [17, 18], then sorted according to developmental stage and sex. Pooling was done on nymphs or male ticks from the same dog while engorged female ticks were placed individually in 1.5 ml tubes. After the addition of 70% ethanol, tick samples were stored at -20 °C until DNA extraction.

### DNA extraction

The extraction of DNA from blood samples was performed using a commercial DNA extraction kit (innuPREP® DNA Mini Kit, Analytik Jena, Jena, Germany) following the manufacturer’s protocol with some modifications in the initial steps. The extraction of DNA from ticks was done using the alkali neutralization method described by Takano et al. [19] with some modifications. Briefly, after removal of ethanol and washing with phosphate-buffered saline, 200 μl or 500 μl of 25 mM NaOH was added directly to pooled nymphs and male ticks or to an engorged female tick, respectively. The ticks were crushed thoroughly using sterile tube pestles, and then the tubes were placed in a boiling water bath for 10 min. After cooling, 16 μl or 40 μl of 1M Tris-HCl was added to nymph and male tick samples or to engorged female tick samples, respectively. The supernatant was recovered after centrifugation at 20,000× *g* for 5 min. All DNA samples were stored at 4 °C until use.

### PCR detection and sequence analysis

Prior to detection of pathogens in the samples, the control genes *actin* and mitochondrial *16S* rRNA (*mt-rrs*) were first detected from blood and tick DNA samples, respectively, through end-point PCR. After positive confirmation of the control genes, nested PCR targeting the *groEL* of *Ehrlichia*/*Anaplasma* spp., and the *18S* rRNA of *Babesia*/*Hepatozoon* spp., was performed. DNA samples that showed positive bands for *Ehrlichia*/*Anaplasma* spp. or *Babesia*/*Hepatozoon* spp. were further subjected to end-point PCR using species-specific primers, while nested PCR targeting the *gltA* gene was also done to detect *Rickettsia*. All of the primers used in this study and their respective annealing temperature and expected product size are listed in Table 1. PCR mixtures (Tks Gflex™, Takara, Shiga, Japan) were prepared following the manufacturer’s recommendation. PCR products were loaded into 2% agarose gel in TAE buffer and then visualized after staining with ethidium bromide. For sequence analysis, selected positive amplicons were purified after excision from the agarose gel using Nucleospin® gel and PCR clean-up kit (Macherey-Nagel, Leicestershire, England) following the manufacturer’s protocol. Additionally, sequencing of the *mt-rrs* from 10 engorged tick samples was performed to further confirm the identity of the ticks. After obtaining the sequence readings, sequences were compared to reported isolates using the Basic Local Alignment Search Tool (BLAST) of the U.S. National Center for Biotechnology Information (https://blast.ncbi.nlm.nih.gov/Blast.cgi).

**Table 1:** Primers used in the detection of various tick-borne pathogens and control genes

Organism	Target gene	Primer name	Sequence (5'-3')	Annealing T (°C)	Product size (bp)	Reference
*Anaplasma*/*Ehrlichia* spp.	*groEL*	gro607F	GAAGATGCWGTWGGWTGTACKGC	57	664	[35]
		gro1294R	AGMGCTTCWCCTTCWACRTCYTC			
		gro677F	ATTACTCAGAGTGCTTCTCARTG	57	315	
		gro1121R	TGCATACCRTCAGTYTTTTCAAC			
*Anaplasma platys*	*groESL*	PLA-HS475F	AAGGCGAAAGAAGCAGTCTTA	63	513	[36]
		PLAT-HS1198R	CATAGTCTGAAGTGGAGGAC			
*Ehrlichia canis*	*gltA*	EcanisFw	TTATCTGTTTGTGTTATATAAGC	53	1372	[37]
		EcanisRev	CAGTACCTATGCATATCAATCC			
*Rickettsia* spp.	*gltA*	CS2d	ATGACCAATGAAAATAATAAT	52	1250	[38]
		CSEndr	CTTATACTCTCTATGTACA			
		RpCs877p	GGGGGCCTGCTCACGGCGG	52	341	
		RpCs1258n	ATTGCAAAAAGTACAGTGAAC			
*Babesia/Hepatozoon*	*18S* rRNA	piro18S F1	GGTGAAACTGCGAATGGCTC	55	1500	This study
		piro18S R1	AAGTGATAAGGTTCACAAAACTT			
		piro18S F2	TGGCTCATTACAACAGTTATA	53		
		piro18S R2	CGGTCCGAATAATTCACC			
*Babesia canis*	*18S* rRNA	BcanisF	GTTTATTAGTTTGAAACCCGC	59	456	[39]
		BcanisR	GAACTCGAAAAAGCCAAACGA			
*Hepatozoon canis*	*18S* rRNA	HepF	ATACATGAGCAAAATCTCAAC	50	666	[23]
		HepR	CTTATTATTCCATGCTGCAG			
Tick control	*mt-rrs*	mt-rrsF	CTGCTCAATGATTTTTTAAATTGCTGTGG	56	460	[40]
		mt-rrsR	CCGGTCTGAACTCAGATCAAGTA			
Blood control	*actin*	Actin-F	CGCACCACCGGCATCGTGAT	65	227	[41]
		Actin-R	TCCAGGGCCACGTAGCAGAG			

*Abbreviation*: *T* temperature

### Data analysis

Based on the information obtained from the questionnaire, dogs were then classified according to age group (puppy, juvenile or adult) and sex. The presenting clinical signs were also noted. After PCR, the detection rate for each pathogen was calculated by dividing the number of positive samples by the total number of samples and then expressed as percentage. The occurrence of TBP infection with regard to dog origin, age and sex was also calculated, and then Chi-square analysis at a 95% confidence interval (α = 0.05) was performed using the online software WinEpi® to determine the presence of association. The detection of multiple pathogens (co-infection) in blood samples was also noted.

## Results

### Data on sample collection

A total of 248 canine blood samples were obtained from 12 locations in southern Metro Manila and Laguna, 198 of which came from a total of 33 participating veterinary clinics and hospitals, while 50 came from a dog pound in Los Baños, Laguna. Although not all dogs had complete information in the provided questionnaires, the available history was still analyzed for grouping according to age and sex. The majority of the sampled dogs were adults (61.3%) and male (56.9%). Not all of the dogs were showing clinical signs at the time of sample collection, but the most common clinical signs indicated in the questionnaire were inappetence, lethargy, fever, pale mucus membranes, bloody diarrhea, epistaxis, hematuria, jaundice and vomiting. Only 90 dogs had ticks at the time of blood collection. A total of 157 tick samples were prepared for DNA extraction after identification and sorting. All ticks were morphologically identified to be *R. sanguineus* (*sensu lato*), 112 (71.3%) of which were females, 27 (17.2%) were males, and 18 (11.5%) were nymphs.

### PCR results

The control genes *actin* and *mt-rrs* were successfully detected in all blood and tick DNA samples, respectively. Following nested PCR using screening primers, the DNA of bacterial pathogens *Ehrlichia*/*Anaplasma* spp. was detected in 56 (22.6%) blood samples, while DNA of protozoan pathogens *Babesia*/*Hepatozoon* spp. was detected in 19 (7.6%) blood samples. The result of species-specific PCR of blood samples that turned positive for screening primers is summarized in Table 2. *Ehrlichia canis* was the most commonly detected TBP (49 dogs, 22.6%), while *Rickettsia* and *H. canis* were the least detected (6 dogs, 2.4%). Analysis of TBP occurrence showed that 61 (24.6%) dogs were positive for a single pathogen, of which the majority were infected with *E. canis* (39 dogs, 15.7%), followed by *B. vogeli* (4%), *A. platys* (2.4%), *Rickettsia* spp. (1.6%) and *Hepatozoon* spp. (1.2%) (Table 3). Multiple pathogens were detected in 14 dogs, which indicate co-infection. Two TBPs of different combinations were detected in 10 dogs (4%), half of which were positive for both *E. canis* and *A. platys*. Interestingly, three dogs (1.2%) were positive for two different combinations of three TBPs, while one (0.4%) was positive for four TBPs.

**Table 2:** Number of positive canine blood and tick samples for tick-borne pathogens (TBPs) that were detected using PCR

TBPs	Blood samples (*n* = 248) (%)^a^	Tick samples (*n* = 157) (%)^a^
*Ehrlichia canis*	49 (19.8)	5 (3.2)
*Anaplasma platys*	15 (6.0)	1 (0.6)
*Rickettsia* spp.	6 (2.4)	0
*Babesia vogeli*	17 (6.8)	1 (0.6)
*Hepatozoon canis*	6 (2.4)	1 (0.6)

^a^Percentages were calculated based on the total number of tested blood and tick samples

**Table 3:** Analysis of occurrence of tick-borne pathogens (TBPs) in dogs based on PCR results

TBPs detected	No. of dogs (%)^a^
1 TBP only	61 (24.6)
*Ehrlichia canis*	39 (15.7)
*Anaplasma platys*	6 (2.4)
*Rickettsia* spp.	4 (1.6)
*Babesia vogeli*	10 (4.0)
*Hepatozoon canis*	2 (1.2)
2 TBPs	10 (4.0)
*E. canis* and *A. platys*	5 (2.0)
*E. canis* and *Rickettsia* sp.	1 (0.4)
*A. platys* and *Rickettsia* sp.	1 (0.4)
*A. platys* and *B. vogeli*	1 (0.4)
*B. vogeli* and *H. canis*	2 (0.8)
3 TBPs	3 (1.2)
*E. canis*, *A. platys* and *B. vogeli*	2 (0.8)
*E. canis*, *B. vogeli* and *H. canis*	1 (0.4)
4 TBPs	1 (0.4)
*E. canis*, *A. platys*, *B. vogeli* and *H. canis*	1 (0.4)
Total number of dogs with TBPs	75 (30.2)

^a^Percentages were calculated based on the total number of tested dogs (*n* = 248)

With regard to the 157 tick samples, 8 (3.2%) and 1 (0.64%) were positive for *Ehrlichia*/*Anaplasma* spp. and *Babesia*/*Hepatozoon* spp., respectively (Table 2). Species-specific PCR showed that *E. canis* was the most detected (5 or 2%) in tick samples. One tick sample was positive for *Ehrlichia*, *Babesia* and *Hepatozoon*. All TBP-positive tick samples were engorged females. *Rickettsia* was not detected in any of the tick samples.

### Occurrence of TBPs with regards to some host attributes

The presence of TBPs in household and impounded dogs in different age groups (puppy, juvenile and adult) and of different sexes was compared (Table 4). A significantly higher number of household dogs presented to veterinary clinics were infected with at least one TBP (37.9%; *χ*^2^ = 12.163, *df* = 1, *P* < 0.001), whereas only 10% of dogs from the dog pound tested positive for any TBP. Chi-square analysis showed a positive association of TBP occurrence with age (*χ*^2^ = 11.082, *df* = 2, *P* = 0.004). The detection was highest in puppies, with 18 of 37 (48.6%) puppies testing positive for at least one TBP. Interestingly, five puppies had concurrent infection with two TBPs, and four were infected with *Rickettsia* sp. The next highest positive detection for at least one TBP was in juveniles (37.3%), and lowest was in adults (26.3%). With regard to sex, more females (37.5%) were found infected with at least one TBP than males (25%), also having a positive association according to Chi-square analysis (*χ*^2^ = 4.473, *df* = 1, *P =* 0.034).

**Table 4:** Occurrence of TBPs with regard to host attributes. Chi-square analysis determined the presence of association, and *P*-values are shown

Attribute	No. of dogs	No. (%) infected with at least one TBP	*P*-value
Origin			<0.001^*^
Dog pound	50	5 (10.0)	
Household	198	70 (28.2)	
Age			0.004^*^
Puppy (< 1 year)	37	18 (48.6)	
Juvenile (1–3 years)	59	22 (37.3)	
Adult (> 3 years)	152	35 (23.0)	
Sex			0.034^*^
Male	144	36 (25.0)	
Female	104	39 (37.5)	

^*^P < 0.05

### Sequence analysis

BLAST analysis of *mt-rrs* from 10 engorged ticks showed that all share 97–100% identity with reported *mt-rrs* sequences of *R. sanguineus* (GenBank: MF351574.1, KC170744.1, AY883868.1)*.* For each genus of TBP detected, at least three positive amplicons were subjected to sequencing and BLAST analysis. All obtained sequences for each pathogen were 100% homologous and were found to share 96–100% identity with reported *E. canis* (GenBank: CP025749.1, KU765198.1, AY647155.1), *A. platys* (GenBank: KY425417.1, AY077621.1, KU765205.1), *B. vogeli* (GenBank: LC331058.1, KU361222.1, KU361220.1) and *H. canis* (GenBank: KC138532.2, AF176835.1, KX818220.1) isolates. For *Rickettsia*, all positive amplicons share 99% sequence identity with the reported isolates of *Rickettsia* spp. such as *R. japonica* (GenBank: DQ909073.1) and *R. raoultii* (GenBank: KR265323.1). Representative sequences were deposited in GenBank under the accession numbers LC428206 (*E. canis*), LC428207 (*A. platys*), LC428209 (*B. vogeli*), LC428208 (*H. canis*) and LC428132 (*Rickettsia* sp.).

## Discussion

The TBPs of dogs investigated in this study have recently become a major focus worldwide because of their significance in canine health and potential zoonotic transmission. Dogs have been in close contact with humans for a long time; thus, humans are at risk particularly for zoonotic TBPs. Furthermore, there is the threat of TBPs becoming established in new geographical areas due to the pet trade, which is made easier through the advent of social media and other online platforms, and increased international mobility [14]. TBPs can cause higher rates of morbidity and fatality in non-endemic areas. In this regard, it is the responsibility of veterinarians to monitor TBPs for effective disease surveillance and to execute strategies that will help eliminate or control the spread of these pathogens. In this study, PCR, which is proven to be highly sensitive, was used in detecting TBPs believed to be common not only in the Philippines but also in other Southeast Asian countries.

Only a few studies have been done on the occurrence of TBPs in dogs in the Philippines. Some of them utilized conventional microscopic examination of blood smears [16, 20]. While this technique is simple and inexpensive, it has been proven to have low sensitivity, especially in finding *E. canis* and *A. platys* [6]. Some serological studies on TBPs have been also conducted [21, 22], and serologically based commercial kits are widely used in veterinary clinics nowadays; however, among the limitations of detecting antibodies is the inability to differentiate past from on-going infection due to persistence of the antibodies.

In the last decade, studies on the molecular detection of TBPs in dogs through PCR have been increasing. However, this generated little epidemiological data in the Philippines because surveys were limited to small areas of the country. Furthermore, previous studies mostly included pet dogs that showed clinical signs and presented in a few veterinary clinics or hospitals. Our current study covered several big cities in Metro Manila, the capital region of the Philippines, as well as cities and municipalities in the nearby province of Laguna, and included both apparently healthy and sick dogs. Additionally, aside from pet dogs brought to veterinary clinics and hospitals, impounded stray or abandoned dogs were included in the study. Because stray dogs are neglected, they play a significant role in maintenance of different TBPs [10, 23–25].

Our results showed that *E. canis* is the most detected pathogen in blood and tick samples. The detection rate of *E. canis* in blood samples obtained in this study is higher than that in previous reports in the Philippines that used PCR, which ranged between 2–10% [11, 14, 15], but is similar to that in the reported detection rate in other Southeast Asian countries [23, 26]. The difference between our results and those of previous studies in the Philippines could be attributed to the difference in the number of dogs and the selection criteria, the geographical area, and the targeted gene. A previous study that utilized a commercial antibody test kit reported 95% seropositivity for *E. canis* in blood of dogs from Metro Manila [21]. Meanwhile, the detection rates for *A. platys*, *B. vogeli* and *H. canis* are close to those in previous PCR-based studies in the country [11, 14, 15].

Another significant finding in this study is the detection of *Rickettsia* sp. in some dogs. *Rickettsia* sp. was detected singly in four dogs and in concurrent infection with species of *Anaplasma* or *Ehrlichia* in two other dogs, all of which are household pets presented to veterinary clinics/hospitals. Unfortunately, the clinical signs were not indicated in the information for those dogs. There are only a few reports on *Rickettsia* infection in dogs in Southeast Asia [26, 27], including the Philippines; hence, very little is known about the occurrence of this zoonotic TBP in the country. Seropositivity to *R. prowazekii*, *R. rickettsii*, and *R. canadensis* has been reported in dogs from Thailand, but none were found positive for any *Rickettsia* spp. using PCR [27]. Recently, *R. felis* was detected in 11 of 101 dogs from Cambodia using nested PCR [26]. There were only two published reports in the Philippines on the detection of antibodies against *Rickettsia* spp. in animals and humans [28, 29]. Camer et al. [28] reported detection of antibodies against *R. japonica* in dogs from Luzon Island of the Philippines. The sequence analysis of *Rickettsia* sp.-positive amplicons in this study suggests that dogs are infected with *R. japonica*. To our knowledge, this is the first molecular evidence for the presence of *Rickettsia* in the Philippines.

All *Rickettsia* species known to infect dogs are zoonotic [9]; hence our findings should raise an alarm because of the risk to humans. Infection with *E. canis* in humans has been also reported, in which *E. canis* DNA was detected through PCR from human patients who showed clinical manifestations of human monocytic ehrlichiosis [30]. Many pet owners in the Philippines keep their dogs indoors, and some are in close contact, even sleeping with their dogs. The risk to humans is augmented by the possibility that *R. japonica* infection in dogs may go unnoticed, as shown by a previous study wherein dogs experimentally infected with *R. japonica* showed non-specific clinical signs, such as fever, anorexia and depression, if immunosuppressed, but not if immunocompetent [31]. Further studies on *Rickettsia* infection in dogs, involving geographical distribution, clinical features, transmission and risk factors, should be performed to promote control of the potential threat to public health.

Concurrent infection with two or more TBPs was fairly common in tested dogs, observed in 22 (8.9%) individuals. The presence of co-infection may result in greater pathogenicity and more complications. Thus, the clinical signs exhibited by the affected dog may be variable [5]. Unfortunately, the incomplete questionnaire returned to us by the participating veterinary clinics/hospitals limited our analysis. Nevertheless, our results emphasize the importance of testing for more than one TBP, especially because the line of treatment for rickettsial and protozoan pathogens is different.

Analysis of some host attributes showed that household dogs, puppies and females have a higher tendency to become infected with TBPs. The majority of dogs included in this study were household pets with existing or a history of tick infestation, which may explain the higher detection rate as compared to dogs from the dog pound, among which only a few had tick infestation at the time of sample collection. Additionally, the brown dog tick *R. sanguineus* is nidicolous and endophilic, preferring to live indoors in close contact with its host [3]. Thus, a household dog staying in one place with a high population of ticks has a higher chance of getting infested repeatedly than does a stray dog that moves from place to place. Nevertheless, stray dogs can be vessels for ticks and spread them from one place to another, thereby playing a role in the spread of TBPs. The higher tendency of puppies to become infected with TBPs could be due to their greater susceptibility to tick infestation and heavier tick burden than older dogs [32]. Our results showed that five puppies were co-infected with two TBPs, which may produce more severe clinical manifestations [33]. The higher detection of TBPs in female dogs could be due to their sedentary habit [34]. Female dogs staying for a long time in areas with high tick population have higher chances of being infested repeatedly with ticks, hence also higher risk of getting infected with TBPs.

## Conclusions

This study showed that *E. canis* is highly endemic in the Philippines and concurrent infection with other TBPs is quite common. To the authors’ knowledge, this study also provides the first molecular evidence of *Rickettsia* infection in dogs in the Philippines. Taken together, these results emphasize the need to test for multiple TBPs in dogs suspected of infection to facilitate appropriate treatment and to raise awareness of the threat to public health. Further investigation is needed to examine the epidemiology and clinical features of rickettsiosis in dogs in the Philippines.

## Abbreviations

BLAST: Basic Local Alignment Search Tool
CME: Canine monocytic ehrlichiosis
TBD: Tick-borne disease
TBP: Tick-borne pathogen.
